# Treating Anxiety-Based Cognitive Distortions Pertaining to Somatic Perception for Better Chronic Pain Outcomes: A Recommendation for Better Practice in the Present Day and the Cyber Age of Medicine

**DOI:** 10.3390/jcm13195923

**Published:** 2024-10-04

**Authors:** Marcelina Jasmine Silva

**Affiliations:** 1 The Focus on Opioid Transitions (FOOT Steps) Program, Walnut Creek, CA 94598, USA; msilva7@touro.edu; 2 The Focus on Opioid Transitions (FOOT Steps) Program, Capitola, CA 95010, USA; 3 Touro University College of Osteopathic Medicine, Vallejo, CA 94592, USA

**Keywords:** chronic pain, catastrophizing, fear avoidance, kinesiophobia, opioids, anxiety, depression, artificial intelligence

## Abstract

Anxiety-based cognitive distortions pertaining to somatic perception (ABCD-SPs)—primarily catastrophizing, fear avoidance, and kinesiophobia—have been repeatedly linked to worsening chronic, non-cancer pain (CNCP) outcomes of increased disability, amplified pain, ineffective opioid use, and opioid misuse. Several studies have suggested that treating ABCD-SPs can improve pain outcomes, yet identification and targeting of ABCD-SPs are not part of the standard medical pain assessment and treatment plan. Utilizing a narrative review of proposed mechanisms, published patient perspectives, and study correlations connecting these cognitive distortions with CNCP outcomes, an approach for better practice in the delivery of standard medical CNCP care can be deduced and formulated into a Belief and Behavior Action Plan (BBAP) for medical clinicians treating CNCP to implement into initial and maintenance care planning. These recommendations require relatively few resources to implement and have the potential to disseminate more effective CNCP treatment on a large scale now and in the future with the new frontier of cognitive computing in medicine.

## 1. Introduction

Chronic, non-cancer pain (CNCP) is estimated to affect more than 100 million adults in the United States [1,2]. The prevalence of this condition is so high that the Centers for Disease Control have declared that efforts to improve the lives of people with CNCP are a public health imperative [1]. Although an array of medical and interdisciplinary pain specialists exist and are trained to offer their respective complementary, interdisciplinary, pharmaceutical, interventional, and surgical pain treatments, CNCP diagnosis and treatment is most often delivered in the primary care setting [3]. Further, there is a disproportionate amount of high-impact pain (defined as pain severity high enough to interfere with activities of daily living) [4] reported in under-resourced and underserved communities that rely upon primary care for all of their medical needs [5].

Limited specialist involvement is only one of the problems within the current CNCP management climate in the Unites States. Recent CNCP treatment standards have resulted in secondary problems for the individual patient, and in the greater public health and managed care arenas. Such problems include ostracizing patient stigma [6,7], morbidity and mortality from adverse medication effects [8], the rise and reign of the opioid epidemic [9], and skyrocketed costs of managed care [7]. The financial burden of morbidity related to CNCP alone is more than that of the afflictions of heart disease and cancer combined, and has been tallied to be over USD 600 billion per year in the US [2].

Identifying more effective and efficient care approaches for those who suffer with CNCP continues to be a priority need in US healthcare, especially approaches that will have enduring relevancy as medicine advances into the cyber age. Artificial intelligence (AI) and machine learning (ML) are rapidly being integrated into everyday healthcare experiences in an effort to reduce healthcare costs [10]. Early estimates of the cost-saving impact of AI use in medicine project a spending reduction between 5% and 10%, saving roughly USD 200 billion to USD 360 billion a year [11]. Improved approaches that can be widely disseminated and implemented in low-resourced areas by the first line of medical clinicians assessing and treating CNCP are urgently needed now. The present time also offers a uniquely primed opportunity in medical history to articulate and integrate these improved approaches into conscious computing medical interfaces. Current efforts to this end have the potential to broaden CNCP treatment accessibility, and to have a resounding effect as the basis for the future of CNCP care.

Pain catastrophizing is the most common cognitive distortion seen in patients with chronic pain, and severe catastrophizing is prevalent for nearly 40% of people experiencing CNCP [12,13]. This belief paradigm has been linked to maladaptive behavior and resulting negative CNCP sequelae [14] exemplified by increased disability [15,16,17,18,19,20,21], pain intensity [22,23], emotional distress [15], absenteeism [19], and ineffective opioid use [22,24]. Cognitive distortions are defined as faulty or inaccurate thinking, perceptions, or beliefs [14]. Catastrophizing is characterized by the belief that the worst possible outcome will occur when in a setting that may be serious and upsetting but is not necessarily disastrous [9]. Pain catastrophizing is associated with feelings of helplessness to succumb to a catastrophic outcome, as well as hypervigilance to behave in a way that avoids stimuli that may insight discomfort in painful areas [25]. The pervasiveness of this symptomatology within the chronic pain experience—in both frequency and influence—identifies it as a target of high relevance when looking to improve the quality of CNCP treatment.

Opioid misuse and ineffective use (referred to in this manuscript as opioid use that does not facilitate adequate analgesic and functional results, or that does not achieve desired medical results that outweigh the burden of adverse medication effects) are contributors to poor patient outcomes and to larger public health concerns regarding the opioid epidemic [7,8,22,23,24,26]. The adverse effects of full mu agonist long-term opioid therapy (LTOT) are numerous and well documented, and are amplified if patients fail to experience reasonable functional and analgesic satisfaction from such therapy. Adverse effects that may occur range from immediate [27] (constipation, dry mouth, cognitive impairment, and abuse liability, potentially fatal respiratory depression, and—in the case of methadone—cardiac arrhythmias [28]) to long term and insidious (hypogonadism [29], immune compromise [30,31,32], and hyperalgesia [33,34]). The chronicity incurred from LTOT use can be burdensome not only to the individual but to society as a whole in the form of increased managed care charges [7], longer lengths of disability [9], and a nationally decreased life expectancy due to fatal opioid-related overdoses [9,35,36,37].

Due to abundant evidence of the negative synergy between pain-related catastrophizing and the morbidity of CNCP, care planning to assess and address this cognitive distortion should be a foundational part of CNCP treatment now and in future digital and cyber iterations of care delivery. Utilizing a narrative review of proposed mechanisms, published patient perspectives, and study correlations connecting this cognitive distortion with disability, pain levels, and/or ineffective opioid use or misuse, an approach for better practice among pain clinicians can be deduced—one rooted in holistic clinical assessment, abundant patient education, supportive fear quiescence, and therapeutic confrontation of concerns. This new approach requires few resources to implement and has the potential to lead to a more effective CNCP treatment on a large scale now and in the future.

## 2. Anxiety-Based Cognitive Distortions Pertaining to Somatic Perception (ABCD-SPs)

More than one assessment scale has been validated in an attempt to quantify the clinical significance of the contribution of pain-related catastrophizing to the morbidity of CNCP. Most of the literature examines the relationship between CNCP sequelae as related to this cognitive distortion via one of the following: the Fear-Avoidance Beliefs Questionnaire (FAB), the Pain Catastrophizing Scale (PCS), and the Tampa Scale of Kinesiophobia (TSK) (Table 1). Due to the plurality of these validated tools, this paper has adopted an encompassing term to discuss the significance of their contributions to the morbidity of CNCP: anxiety-based cognitive distortions pertaining to somatic perception (ABCD-SPs).

### 2.1. An Overview of the Role of ABCD-SPs in the Negative Sequelae of CNCP

ABCD-SPs in the setting of CNCP have been repeatedly linked to worsening pain outcomes. Such beliefs, and resulting maladaptive behaviors, have been associated with increased disability [15,16,17,18,19,20,21], pain intensity [22,23], emotional distress [15], and absenteeism [19]. Studies have shown that fear of movement and reinjury is a better predictor of self-reported disability and treatment failure than biomedical findings, or pain intensity levels [43,44,45]. ABCD-SPs have also been documented to affect opioid use in terms of prolonging postoperative use, increasing opioid craving, and contributing to general misuse [22,46,47,48,49].

Objectively, improvements in ABCD-SPs can be visualized on functional MRI, and improvements correlate with a decreased pain state [50,51]. Catastrophizing has been shown to recruit regions of the brain that evoke a more intense suffering response to mild pain and an inability to decouple and suppress more intense pain when compared to controls [50]. A successful decrease in catastrophizing via cognitive behavioral therapy (CBT) has been shown on functional MRI to increase the mass of a subject’s gray matter—an anatomical substance known to generally be reduced in volume and density in patients who suffer with chronic pain [51].

Perhaps most persuasive regarding the import of ABCD-SPs to CNCP-related morbidity are the studies that suggest treating ABCD-SPs can reverse some of the negative sequelae associated with CNCP. It has been documented that treatment campaigns targeting ABCD-SPs can have a positive effect on the clinical outcomes of somatic symptom prevalence and the length of pain episodes when effectively reduced [38,52,53,54]. Some studies have shown efficacy in harnessing ABCD-SP education to affect positive change in disability length related to CNCP [52,55].

### 2.2. ABCD-SP Validated Assessment Tools

#### 2.2.1. The Fear-Avoidance Beliefs Questionnaire (FAB)

The Fear-Avoidance Beliefs Questionnaire (FAB) was designed to measure fear-avoidance beliefs about physical activity and work, and it has strongly correlated these beliefs with work loss and pain [16]. The FAB consists of two subscales: Work (FAB-W) and Physical Activity (FAB-PA). Several studies have investigated the reliability of the FAB for the assessment of fear avoidance among patients with various etiologies of CNCP [38,43,56,57,58]. A higher FAB score has consistently been shown to correlate with an increased probability of current and future work loss and disability [16,19,20], as well as social withdrawal [21]. While the relationship between an elevated FAB score and increased disability and pain remain correlated, the optimal cut off for determining a significant FAB score in relation to negative chronicity in CNCP has varied with the pain context [16,38,52,56,58,59,60]. Higher FAB scores have also been shown to significantly predict treatment failure [56,57]. FAB analysis has also been used to determine which clinical interventions have a better likelihood of a successful outcome to decrease patient-reported disability and pain [56,57,58,61]. An elevated FAB-PA has been shown to be a strong correlate with the inability to cease ineffective LTOT use, more so than morphine equivalent levels and elevated Current Opioid Misuse Measure scores [22].

Several studies have examined the relationship between improved disability and treatment of CNCP via graded exposures that confront fear-avoidant beliefs and behaviors to improve patient self-efficacy and overall disability [62,63,64,65,66,67,68]. FAB-targeted educational campaigns have had positive effects on beliefs and clinical outcomes [38,52,53,54]. Specifically, one study found that successfully lowering fear-avoidance scores in patients with chronic back pain through an educational campaign resulted in subsequently decreased patient reports of disability [52].

#### 2.2.2. The Pain Catastrophizing Scale (PCS)

The PCS determines a patient’s level of pain catastrophizing, which is tested by assessing the elements of rumination, magnification, and helplessness [42]. It was created to better assess the relationship between greater pain intensity, negative pain-related thoughts, and greater emotional distress. Higher scores have been shown to significantly correlate with a prediction of pain intensity and emotional distress [42,46,48,55,57,69], and have also been implicated as a risk factor for increased disability length [48,54,58], pain interference [69], and delayed return to work [41]. Preoperative catastrophizing can even predict higher postoperative pain levels and poorer patient-reported postoperative satisfaction following minimally invasive implantations [70] and surgery [71,72,73,74]. It has been postulated that this correlation may contribute to increased use of healthcare services, and higher costs to the healthcare system [75].

Targeted therapy to improve catastrophizing has been shown to significantly improve pain outcomes. Pain intensity and disability have been shown to improve with improved PCS scores when maladaptive beliefs were challenged via education and cognitive restructuring, even when such interventions occurred on a purely theoretical and cognitive level [62]. Combined physical therapy (PT) with treatment to minimize psychological catastrophizing barriers improves return-to-work rates [55,66]. One study reported this treatment combination had a 25% higher return-to-work rate than physical therapy alone [55]. PCS score improvements have also been correlated with successful cessation of ineffective LTOT in a population for whom cessation had not been previously achievable through usual care methods [23].

#### 2.2.3. The Tampa Scale of Kinesiophobia (TSK)

The TSK is a measure of fear of movement, injury, or reinjury [39] and has been validated for use in assessing comorbidities of chronic pain from multiple etiologies including backpain, neck pain, lower-limb complaints [76,77], and fibromyalgia [17,39,78]. Increased TSK scores are implicated in decreased physical performance and increased pain intensity, depressive symptoms, pain-related anxiety, and disability [17,79]. Like the PCS and FAB, the TSK has also been associated with ineffective opioid use and misuse [22,23,24]. Several studies have shown that high kinesiophobia is an independent risk factor for less satisfactory treatment outcomes [76,77,80]. Also, similar to the other ABCD-SP assessments, studies show that targeted cognitive exercises for decreasing kinesiophobia can improve disability [80] and pain [81] when combined with PT better than PT alone [82,83,84], and can improve pain intensity and physical function [85,86,87].

## 3. Pathology of Anxiety-Based Cognitive Distortions Pertaining to Somatic Perception—Proposed Mechanisms

**Figure 1:**
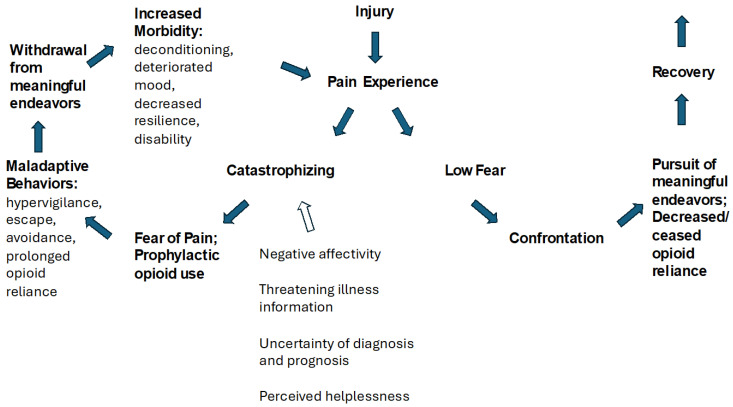
The Fear-Avoidance Belief and Behavior Model [16,22,56,57].

The Fear-Avoidance Belief and Behavior Model (Figure 1) [16,22,56,57] can be visually represented to illustrate the different trajectories for patients with a low fear reaction versus patients with a catastrophizing reaction to their pain experience. The basic tenet of the model is that the way in which pain is interpreted leads to two potential pathways. When pain is perceived as non-threatening, or low threat, patients are likely to behave in a way that confronts real, or perceived, factors that limit their pursuit of meaningful endeavors. This step of confrontation is imperative to eventually overcome those limitations and move toward recovery. In the case of opioid use, the low-fear pathway leads to the use of only a short course of opioids before decreasing use, or ceasing use altogether, thus minimizing or eliminating adverse medication effects [22].

In contrast, a maladaptive cycle may be initiated when pain is perceived through a catastrophizing lens. Catastrophizing entails, among other things, a sense of feeling overwhelmed and powerless to succumb to external, negative forces and experiences [14,62]. This gives rise to pain-related fear, activity avoidance, experience escape (including prolonged opioid use, or misuse), and a negativity-biased hypervigilance. These propensities lead to a progressive withdrawal from meaningful activities and an eventual decline in the physical and emotional capability to access resiliency-building experiences and tools, as previously identified activities of meaning become less attainable. While avoiding the stimuli suspected, or proven, to provoke pain can be adaptive in the acute pain stage, it paradoxically entrenches disability and reliance upon opioids in the subacute and chronic stages of pain. Eventually, the long-term consequences of deconditioning due to disuse [88] and mood deterioration [26,46,89,90,91] result in increased morbidity [9,36] and decreased ability to recruit and access alternative, resilience-building chronic pain coping mechanisms [22].

Several things can accelerate and amplify the maladaptive cycle. Receiving threatening information about a diagnosis can understandably send a patient’s focus to worst-case scenario possibilities. However, uncertainty about a diagnosis can be just as disturbing as threatening information [16,92]. Lack of understanding about the significance of pain is one of the main reasons patients with CNCP go to the Emergency Department (ED) [93]. Negative affectivity and mood disorders, especially anxiety and depression [94,95], coexisting in the patient promote and propel the catastrophizing cycle via a distortion of negativity bias [92]. Also, a history of trauma—even if it precedes the inciting pain event—can propel this maladaptive cycle. A large body of evidence shows that numerous morbidities are accelerated, if not generated, by exposures to adverse childhood experiences (ACEs) [96]. ACE exposure fosters general hypervigilance and negativity bias in daily experiences, resulting in increased catastrophizing and pain-related suffering, among multiple other poor health outcomes [97].

Fear avoidance of movement due to pain, from the stance of learning theory, is a self-perpetuating dynamic in which a small sensory insult—or even the threat of such an insult—can propel anticipation of hyperbolized potential consequences. This anticipation can create—and reinforce—global, habitual, and maladaptive behavior [98], and hinder trials of adaptive activity. If the expectation of catastrophic pain is not confronted, it cannot be disproved. This leads to further maladaptive beliefs and behaviors, deconditioning, and disability [16,22,99,100]. As Vlaeyen et al. state in their paper describing the Fear-Avoidance Model, “Avoidance can be used as a source of information to derive danger, for example: “I am avoiding, therefore there must be danger”. The relief that the expected threat did not occur may reinforce avoidance behavior, and hence maintain it [92].

This uninterrupted cyclic dynamic is also applicable in the context of problematic LTOT usage, as many patients associate the action of taking a scheduled opioid with that of prophylactically avoiding or escaping pain. In this pattern, the unadulterated experience of physical nociception is rarely confronted, and patients can spiral deeper into habitual opioid administration, and the possible adverse effects of LTOT use. This dynamic is compounded in opioid use, as it is triply reinforced by dopaminergic incentivization and abrupt abstinence syndrome disincentivization [22].

## 4. The Call for a Belief and Behavior Action Plan—Theoretical Considerations

**Figure 2:**
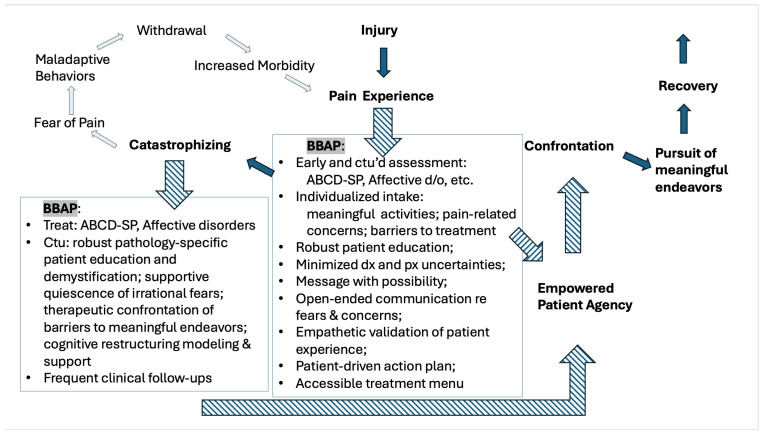
Belief and Behavior Action Plan (BBAP) for CNCP: The better-practice treatment approach is recommended to be inserted by clinicians at specifically identified care plan intervals—indicated by striped arrows—in order to shunt treatment outcomes toward more adaptive outcomes. Abbreviations: ctu/ctu’d = continue/continued; d/o = disorders; dx = diagnostic; px = prognostic.

Using a reverse-engineering approach to the Fear-Avoidance Belief and Behavior Model, entry points for promoting a more healthful ABCD-SP dynamic in the pursuit of better CNCP outcomes emerge for the pain clinician (Figure 2). The maladaptive cycle is amplified and accelerated when the patient perceives threatening illness information, uncertainty of diagnosis and prognosis, and the perception of powerlessness to succumb to an overwhelming amount of negative sequelae. Thus, initial and ongoing quality communication between a medical clinician and patient about pain etiology, treatment, and prognosis is substantive to the patient’s pain experience and treatment outcome potential. Much as the technique of motivational interviewing has been a highly effective and relatively easy treatment technique to disseminate to improve significant measures in multiple chronic physical and psychological diseases [101], there is an opening for a simple—yet sophisticated—change in clinicians’ approach to communication with patients experiencing CNCP. Specifically needed is a patient-centered, individualized approach to treatment planning that develops empowered agency and supports therapeutic ABCD-SP identification and confrontation within the scope of patient-identified endeavors of meaning. This treatment approach should address patient-disclosed fears, concerns, and misconceptions in a supportive, open-ended, and ongoing manner. The goal should be to culminate the clinical visit with a patient-specific Belief and Behavior Action Plan (BBAP) for CNCP treatment.

To begin to formulate what a BBAP for CNCP would entail, we must first look beyond data and diagrams to the patient perspective. Numerous reports have documented patients’ dislike of—and frank objection to—medical discussions involving “catastrophizing” and like terms. Many patients have called the concept of categorizing their pain experience in this way—as a maladaptive psychological response and behavior—as condescending, and even disenfranchising [102]. Some feel that validated terms currently used within the medical community to assess and address ABCD-SPs carry connotations of “pain shame” [103]. Patients have reported that the label of “catastrophizer” is perceived as unempathetic, stigmatizing, blaming, judgmental, dismissive, minimizing, and weaponizable as a tool to selectively restrict treatment [102]. Some have even contended that the term “catastrophizing” can harbor systemic racism and microaggression, especially when a care plan has failed to distinguish between an ABCD-SP and a generalized stress response to the overall institution of medicine, which for some is a construct fraught with inaccessibility [104], injustice, inequality, and discrimination [105,106]. Some scholars have set about renaming the phenomena of catastrophizing altogether [107]. Despite the mounting volume of these valid and important voices, many feel resigned that stigma will eventually undermine any nomenclature revision attempt to create a patient-centered term used to describe the ABCD-SPs that are a prominent feature in the CNCP experience, and that the stigma lies with the way that people categorize the phenomena [108].

Semantically, the notion of categorizing ABCD-SPs experienced by so many with CNCP as an added pathology is arguably redundant, if not excessively persecutory—a point that has also been made by patients and advocacy groups [102,103]. Pain is defined as, “An unpleasant sensory and emotional experience associated with actual or potential tissue damage, or described in terms of such damage” [109]. Literally, the emotional experience associated with potential tissue damage is real pain, and consequently, treating the ABCD-SP should be conceived of as providing holistic pain care. Thus, ABCD-SPs should be evaluated for, and addressed, like other associated pain symptoms and features—such as radicular symptoms or paresthesia—in every case of CNCP. Each of these features should be associated with the appropriate treatment line item in a comprehensive care plan, just as a different medication class might be used for neuropathic versus axial pain.

The above discussion begins to illuminate the need for a paradigm shift in the conceptual construct of not only the patient but also the medical provider. First and foremost, patients need to be approached with empathy. While this may seem self-evident, empathy is not a universal patient experience for patients with CNCP, who have likely experienced diagnosis-associated discrimination and marginalization from the general and medical communities [110]. Aside from maintaining the integrity of the Hippocratic Oath, empathy and trust are imperative to bring about fertile ground for true cognitive, physical, and prognostic change for patients with CNCP [111]. While it is difficult for clinicians to focus on a patients’ suffering because of the accompanying sense of clinical impotence, and frequent lack of objective solutions, simply witnessing the patient’s subjective suffering experience may decrease suffering in itself [112]. Further, in a cohort study that included 1470 adults with chronic lower back pain, physician empathy was more strongly associated with favorable outcomes pertaining to pain, function, and quality of life than were nonpharmacological treatments, opioid therapy, and lumbar spine surgery [113]. This approach bears particular portent in the contemporary environment where many patients have become “opioid refugees” [114,115,116]. This dynamic is compounded for populations experiencing generalized discrimination due to racial, ethnic, gender, or sexual backgrounds or identities that differ from that of their clinician [117,118,119,120].

## 5. Creating a Belief and Behavior Action Plan—Clinical Considerations

Creating a comprehensive and empathetic BBAP for CNCP begins with thorough information gathering on the part of the clinician. An effective pain evaluation and assessment must go far beyond the “OPQRST” (Onset, Palliation/Provocation, Quality, Radiation, Severity, Timing) that is taught in training. It should include a comprehensive mood assessment as well as a healthcare literacy reconciliation between what the patient has been told and what the patient understands—and believes—about their diagnosis and prognosis. Also included should be a cataloging and recording of the patient’s pain-related concerns; a recollection of the patient’s similar past experiences; and adequate time to discuss expectations about potentially affected patient-identified meaningful activities. A thorough clinician will also be cognizant of a potential history of actual or perceived disenfranchisement, discrimination, or implicit bias on the part of the medical system toward the patient [119,120]. It has been postulated that inquiring about this last experience openly and early may help avoid repeat offenses on the part of unintending clinicians, and facilitate a more equitable and effective therapeutic relationship [105]. Any, and all, of the factors above—and potentially more—can result in anxiety that can ignite and fuel the fear-avoidance belief and behavior cycle [18,92,105], and each symptom—and associated belief and behavior—should be documented, triaged, and revisited every visit as part of the symptomatology requiring palliative and restorative CNCP care planning.

The goal of the BBAP for CNCP should be to end with a patient-empowering care plan strongly rooted in patient self-efficacy. The patient and clinician should work together to create a menu of patient treatment options, independent of the part of the care plan that relies upon a medical, rehabilitative, or behavioral health clinician, which should not be usurped by the efforts invested in the making of the BBAP. To assign the appropriate patient-administered actions to the BBAP, the patient’s descriptions of symptoms should be cataloged in terms of levels of severity and physical and emotional distress, along with an associated detailing of the default patient behavioral reaction to these symptoms. These scenarios should then be examined individually and optimized when effective, and gently challenged and replaced when they have been ineffective in the past. The end result is that the patient should leave every clinical visit with an approachable and navigable treatment action plan documenting several ways in which they have control and agency to access pressure release valves for the full spectrum of pain flare severity that may occur. These BBAP interventions should include features that are accessible when in and out of the home, and which represent treatment modalities from a variety of dimensions, including behavioral, physical, social, medical, spiritual, and occupational.

## 6. Creating a Belief and Behavior Action Plan—Recommendations and Practical Considerations

The following is a synopsis of clinical and practical better-practice recommendations to create a BBAP for CNCP, derived from current evidence (Figure 2):

- Utilize standardized assessments and short-answer questionnaires upon initial evaluation, and periodically in follow-up, to assess and monitor the potential for ABCD-SPs to interfere with rehabilitation:
  - Standardized assessments:
    - Assess for high ABCD-SP via one of the frequently used, validated assessments (FAB, PCS, or TSK) [16,22,24,42,57,92,102].
    - Assess for mood disorders that can be independent risk factors for ABCD-SP escalations, especially depression and anxiety [22,24,94,95].
    - Offer a validated instrument assessing perceived discrimination and/or trauma history [96,105,121].
  - Short-answer questionnaires to catalog patients’ perceptions regarding the following:
    - Concerns and fears related to their pain or treatment [57,92].
    - Perceived barriers to accessing helpful pain treatment [104,122].
    - Activities of meaning, which can help accomplish the following goals:
      1. Lay the groundwork to create an individualized care plan.
      2. Strategize support and diminish negative impact on these activities.
      3. Better motivate patient participation [24].
      4. Apply to cognitive restructuring exercises [57,92].
      5. Aid in decreasing treatment plans rooted in implicit bias for populations heralding from a race, culture, gender, sexuality, or age group that differs from the provider [123].
    - Satisfaction with their current and previous pain treatment, e.g., which interventions, medications, therapies, etc., have been perceived as the most helpful, which were the most problematic and why [22,24].
- Implement an intentional BBAP inquiry and communication strategy and style in the clinical visit:
  - Invest heavily in the first visit by performing a deep exploration and inquiry into the patient’s pain experience and their current pain-related beliefs and resulting behaviors (much of which can be initiated via the short-answer format suggested above) [101].
  - Demonstrate empathy [113,124].
  - Use validating active listening, which has been shown to increase patient adherence to care planning [125].
  - Lean into, and address head-on, patient’s accounts of suffering and fear in the clinical setting to achieve the following:
    - Dispel the ability of these sentiments to hijack adaptive recovery processes when the patient ruminates alone [93].
    - Decrease the suffering of invisibility that patients with CNCP often face. While it is difficult for clinicians to focus on a patients’ suffering because of the accompanying sense of clinical impotence, and frequent lack of objective solutions, it has been suggested that simply witnessing the patient’s subjective suffering experience may decrease the severity of the same suffering [112].
  - Be cognizant of both the implicit and explicit messaging inherent in communications imparted by the clinician to the patient about diagnosis and prognosis. Positive self-perceptions and health-related optimism correlate with improved pain suffering, pain-related disability [92,95,97,126,127,128], and even increased longevity [129]. When possible and appropriate, choose vocabulary and descriptors that de-escalate the patient’s perceived threat of nociceptive input, and which highlight functional and meaningful possibility.
  - Message with mindfulness of potential emotional trauma-affected hyperarousal and increased sensitivity to pain [130].
  - Temper areas of diagnostic uncertainty and remaining investigation with clear descriptions of the investigative next steps, while explicitly outlining the activities that are safe to pursue in the interim [57,92].
  - Increase healthcare literacy and promote pathology demystification:
    - Ask patients to paraphrase their understanding of their injury, pain, and pathology. Note terminology used and connect medical terminology to patient’s perceptions and descriptions to promote demystification [57,92]. Correct misconceptions while maintaining patient-generated frame of reference and terminology, when appropriate.
    - Consider inviting a call and paraphrased repeat opportunity between the clinician and the patient to improve comprehension of pathology and related care plan.
    - Assuming the standard use of language interpreters to bridge translation barriers, also employ visual aids and physical models to engage multiple patient learning style preferences to explain not only pathology but also the mechanisms of pain symptomatology in an effort to demystify and decrease anxiety related to somatic perceptions.
  - Orient to when fear of catastrophe is warranted.
    - Debrief previous urgent, or emergent, clinical visits to seek pain treatment. Discuss causational factors and a care plan for future episodes in the form of improved medication organization, strategized BBAP interventions, a change in medication regimen for more effective analgesia, a change in formulary or treatment type for improved access, etc.
    - Orient to “red flag” signs and symptoms that medically warrant emergent attention and educate to differentiate from chronic, stable stimuli.
- BBAP components should include the following:
  - Cultivate an empowering, patient-driven action plan (to complement the encompassing medical and interdisciplinary treatment plan) containing the following elements:
    - Facilitation of a menu of active, self-care options to address various pain levels and flares. Include features accessible in and out of the home, and which represent treatment modalities from a variety of psychosocial domains: behavioral, physical, social, medical, spiritual, occupational, etc.
    - Minimized barriers and avoidance of the “gate keeper” perception of clinical treatment options where possible—within the confines of evidence-based care—which inherently promote a role of helplessness, perceptions of scarcity, and an external locus of control. Instead, promote care planning options that are autonomously administered and are rooted in patient agency, including the following:
      1. Prescribe medications and self-administered devices that can be safely used as needed for specific indications [22,24].
      2. Orient to a home exercise program that de-amplifies pain suffering via an assortment of activities rooted in multiple psychosocial domains [62].
      3. Plan a care regimen creatively, and individually, around potential socioeconomic barriers to treatment access (transportation, mobility, coverage, cost, etc.) by choosing generic medications, refilling for longer durations, providing telemedicine, etc. [131].
  - Use a patient-controlled mechanism to maintain a continuous log of worries and fears associated with pain symptomatology in the CBT-based exercise of cognitive restructuring [132], which has been shown to be helpful for CNCP outcomes even when self-administered [133,134].
  - Carry out frequent routine clinical follow-ups to consistently support the ABCD-SP cognitive restructuring process in the model of treatment recommended for somatoform disorders [135,136], as catastrophizing and somatoform disorders share many clinical features and frequently co-exist [137].

## 7. Discussion

### 7.1. Anticipated Objections to BBAP Implementation—Financial Disincentivization

Clinical efficiency and the demands of billable time have been cited as a barriers to more encompassing CNCP care planning [138]. Cultivating a BBAP that fosters patient empowerment and autonomy, and adequately addresses patient-specific healthcare literacy and individualized concerns, requires time and resources that clinicians are often disincentivized to employ during their limited billable minutes. However, this nearly ubiquitous impediment of limited clinical facetime stems from an unbalanced and short-sighted cost–benefit equation. A minimally invasive surgery or procedure may take as much time—and reimburse exponentially better—than a thorough face-to-face conversation with a patient seeking palliation for a pain complaint. Similarly, a clinician can complete several billable prescription refill visits in the time it takes to thoroughly communicate with one patient. The ironic counterproductivity of this dynamic is illustrated by research that suggests that patients will be less satisfied with the outcomes of these same interventions [70], surgeries [71,72,73,74], and medications [22] if their ABCD-SPs are not adequately addressed first. Thus, a dynamic of high prescription and procedural implementation has been created and perpetuated, often accompanied by dissatisfaction with treatment outcomes. Ultimately, attempts to conserve clinician resources by delaying holistic pain care rooted in emotional resiliency building, stress reduction, and health education in a human-centered approach has resulted in higher overall managed care costs regarding patients with CNCP [7].

Further, much like incorporating motivational interviewing has been found to have a favorable cost–benefit ratio for treatment outcomes of various etiologies in multiple medical settings [139,140], implementing a potentially more efficacious treatment that simply involves a change in communication strategy, such as a BBAP, requires very little resource investment. Considering the current financial and opportunity costs inherent in the standard care approach to treating CNCP in the US, incorporating a BBAP would only require a relatively small investment in medical clinician training hours. Additionally, a BBAP is a care planning strategy that can equally serve populations that are abundantly resourced or under-resourced, alike, potentially bridging some of the inequities currently seen in populations who disproportionately suffer with high-impact CNCP.

### 7.2. Anticipated Objections to BBAP Implementation—Scope of Practice Creep

The significant role that psychologists and allied professionals contribute to the myriad facets of CNCP treatment via an interdisciplinary care plan is not meant to be replaced, or undermined, by the recommendation for the pain clinician to create a BBAP. In fact, most studies have shown greatest success when addressing ABCD-SPs via multimodal efforts, especially when including physical therapies, CBT, and/or acceptance and commitment therapy [141]. However, CNCP is frequently associated with—and compounded by—limited access to such resources [131]. Thus, the recommendation to implement a BBAP for CNCP is non-exclusive in regard to behavioral health specialist collaboration, and is designed to benefit from the more in-depth and expansive behavioral health treatment that a specialist in that field can provide, if accessible.

Further, a BBAP has the potential to champion interdisciplinary care offered by available behavioral health clinicians. Patient buy-in of behavioral health treatment is often improved when medical clinicians specifically endorse and provide education to help patients better understand the far-reaching implications of behavioral health efforts in their medical treatment and recovery [142]. Also, removing some of the figurative partition siloing the physical from the psychological symptoms and treatment of CNCP can help decrease the stigma of psychological suffering related to CNCP, which has the potential to improve outcomes, as described above.

### 7.3. BBAP Relevance and Potential: Medicine in the Cyber Age

Artificial intelligence (AI) and machine learning (ML) are rapidly being integrated into everyday healthcare experiences in an effort to reduce healthcare costs [10,11]. Harnessing the potential for improved treatment outcomes via a BBAP-informed AI algorithm could augment healthcare savings even further in the costly field of CNCP by enhancing outcome improvement and increasing spending returns, not just reducing healthcare costs. It is imperative that the medical community lays a foundation of effective and human-centered design into patient interfaces, as they may be programed into conscious computing perpetuity. This is a uniquely primed time in history to consider which strategies are working well to deliver preferred medical outcomes, and which need improvement, as we enter this new cyber frontier of medical practice.

The integration of cognitive computing in healthcare presents an interesting potential for advancement in crafting personalized medical and psychological treatment plans, and to improve patient and clinician satisfaction [10,11]. Artificial intelligence (AI) can employ algorithms to process standardized screening assessments and generate comprehensive treatment strategies that address both medical and psychological aspects of patient care. AI algorithms are already supporting clinical decision making in many medical disciplines [143] and have the potential to streamline the holistic assessment recommended in a BBAP to drive more efficient, effective, and individualized care planning. Natural language processing (NLP), machine learning (ML), and specified design principles have the potential to customize the patient interface while adhering to a consistent communication strategy, congruent with that of a BBAP. All of this has the potential to be available to adapt and respond with the real-time needs of the patient.

Once again, one can look to the example of motivational interviewing to envision the potential of BBAP propagation in the cyber age. AI is being successfully used to train physicians to better demonstrate the technique of motivational interviewing in their patient visits [144]. This suggests that AI may be a promising avenue of disseminating continuing medical education for BBAP implementation. Programmers and AI developers are also harnessing AI, NLP, and ML to perform digital and AI-assisted motivational interviewing [145,146,147]. It follows that these same cyber techniques could successfully generate and support the individualized, human-centered design of a BBAP.

## 8. Limitations

The aim of this article is to utilize a narrative review of currently available evidence and observations to recommend a better-practice approach to CNCP care, as chronic pain management is an area identified as being in great need of improvement in terms of related public health, individual medical outcomes, and national financial impact. A recommendation for better practice based on such observations has the inherent limitations of not being directly tested or proven as an intervention, as would be the gold standard. Also inherent in the limitations of validating the benefits of a BBAP is the fact that the nature of a BBAP is highly individualized so as to be nearly universal in its applicability. Standardizing a randomized controlled trial for such an approach would be challenging. Future research is encouraged in the form of initial case studies and pilot programing to better understand the impact possibilities of BBAP implementation.

## 9. Conclusions

Due to abundant evidence of the negative synergy between ABCD-SPs and worsening sequelae of CNCP, care planning to assess and address ABCD-SPs via a BBAP should be a foundational part of CNCP treatment. While a multidisciplinary approach is ideal, the role of the individual pain clinician is poised to have a profound effect on a patient’s formation—and either maintenance or dissipation—of ABCD-SP, which is a determinant of CNCP severity and morbidity. CNCP is a multifaceted bio-psycho-social diagnosis, and treatment requires a complex, holistic approach. Maximizing every treatment avenue available is imperative to improve CNCP-related outcomes on the individual and public health fronts. Utilizing a better-practice BBAP is a low-risk, low-investment intervention that currently has the potential to yield high gains on individual and public health levels, and is a strategy that also may be of high relevance in the cyber age of medicine.

## Figures and Tables

**Table 1:** Table of Assessments for anxiety-based cognitive distortions pertaining to somatic perception (ABCD-SPs).

Fear-Avoidance Beliefs Questionnaire—Work and Physical Activity (FAB-W and -PA) [16,22,38]	Two subscales (FAB-W: 0–42; FAB-PA 0–24) in which higher scores indicate more severe pain and disability due to fear-avoidance beliefs about work and physical activity, respectively. Various score thresholds have been documented as associated with clinical relevancy and specific negative chronicity of CNCP. Higher scores have been associated with poor physical and manual therapy results and low return-to-work rates after an injury.
Tampa Scale of Kinesiophobia (TKS) [39,40]	A measure of fear of movement and reinjury. Scores range from 17 to 68, with higher scores being of higher severity. Higher TKS scores have been correlated with higher disability and pain scores.
Pain Catastrophizing Scale (PCS) [24,41,42]	Assesses levels of catastrophizing. In initial validation, a score of 30 or more correlated with high unemployment, self-declared “total” disability, and clinical depression. However, various lower score thresholds have been documented as associated with clinical relevancy for specific negative chronicity of CNCP.
